# Recovery of a Bacteriocin-Like Inhibitory Substance from *Lactobacillus bulgaricus* FTDC 1211 Using Polyethylene-Glycol Impregnated Amberlite XAD-4 Resins System

**DOI:** 10.3390/molecules25225332

**Published:** 2020-11-16

**Authors:** Nur Fazrin Husna Abdul Aziz, Sahar Abbasiliasi, Zhang Jin Ng, Mazni Abu Zarin, Siti Nurbaya Oslan, Joo Shun Tan, Arbakariya Bin Ariff

**Affiliations:** 1 School of Industrial Technology, Universiti Sains Malaysia, Gelugor, Pulau Pinang 11800, Malaysia; fazaziz93@gmail.com (N.F.H.A.A.); jinng@student.usm.my (Z.J.N.); mazniabuzarin@gmail.com (M.A.Z.); 2 Halal Products Research Institute, Universiti Putra Malaysia, Serdang 43400, Selangor, Malaysia; sahar@upm.edu.my; 3 Institute of Bioscience, Universiti Putra Malaysia, Serdang 43400, Selangor, Malaysia; 4 Department of Biochemistry, Faculty of Biotechnology and Biomolecular Sciences, Universiti Putra Malaysia, Serdang 43400, Selangor, Malaysia; snurbayaoslan@upm.edu.my; 5 Bioprocessing and Biomanufacturing Research Centre, Faculty of Biotechnology and Biomolecular Sciences, Universiti Putra Malaysia UPM, Serdang 43400, Selangor, Malaysia; 6 Department of Bioprocess Technology, Faculty of Biotechnology and Biomolecular Sciences, Universiti Putra Malaysia UPM, Serdang 43400, Selangor, Malaysia

**Keywords:** purification, bacteriocin-like inhibitory substance, lactic acid bacteria, polymer, resin

## Abstract

*Lactobacillus bulgaricus* is a LAB strain which is capable of producing bacteriocin substances to inhibit *Staphylococcus aureus.* The aim of this study was to purify a bacteriocin-like inhibitory substance (BLIS) produced by *L. bulgaricus* FTDC 1211 using an aqueous impregnated resins system consisting of polyethylene-glycol (PEG) impregnated on Amberlite XAD4. Important parameters influencing on purification of BLIS, such as the molecular weight and concentration of PEG, the concentration and pH of sodium citrate and the concentration of sodium chloride, were optimized using a response surface methodology. Under optimum conditions of 11% (*w*/*w*) of PEG 4000 impregnated Amberlite XAD4 resins and 2% (*w*/*w*) of sodium citrate at pH 6, the maximum purification factor (3.26) and recovery yield (82.69% ± 0.06) were obtained. These results demonstrate that AIRS could be used as an alternate purification system in the primary recovery step.

## 1. Introduction

*Staphylococcus aureus* skin infections pose a major concern to public health, largely owing to the steadily increasing prevalence of drug resistant isolates [1]. As an alternative mode of treatment, bacteriocins have been shown to possess antimicrobial efficacy against multiple drug resistant strains. This reveals the huge potential of bacteriocin as an alternate drug in the pharmaceutical industry. Bacteriocins are ribosomally synthesized, antimicrobial peptides or proteins produced by various microorganisms which have the ability to inhibit the growth of bacteria with closely related species. The most studied bacteriocin-producing microorganisms are lactic acid bacteria (LAB), which have the status of generally recognized as safe (GRAS) microorganisms [2]. *Lactobacillus bulgaricus* is a LAB strain which is capable of producing bacteriocin-like inhibitory substances (BLIS) to inhibit *S. aureus* [3]. Hence, adequate purification of BLIS from *L. bulgaricus* is necessary for its characterization and potential industrial application.

Different methods have been applied for the recovery of BLIS from fermentation broths. The most frequently used methods for its isolation, concentration, and purification include salt precipitation, various combinations of gel filtration, different chromatography techniques and aqueous two phases [4,5,6]. Most of these methods produce satisfactory results only on a small scale, i.e., with low yields. Furthermore, they are expensive, difficult to handle and not applicable on a large scale [7]. The increasing demand for natural products with bioactive properties calls for new approaches in the biomanufacturing of such products. However, the limiting factor towards their large-scale production has always been the absence of a suitable process to overcome the limited product recovery. In this respect, recent advances in biotechnology have been looked upon as alternative approaches to surmounting this constraint.

Aqueous impregnated resin system (AIRS) is a purification technique for the separation, extraction and concentration of biomolecules. So far, AIRS has only been tested on the purification of enzymes such as esterase and lipase [8,9]. This study evaluated the feasibility of AIRS for the recovery of BLIS from the culture of *L. bulgaricus* FTDC 1211. As far we know, this is the first study on the purification of a peptide like BLIS using Amberlite XAD4 in AIRS. The effects of influencing factors such as the molecular weight and concentration of PEG, concentration and pH of extraction solution (sodium citrate) and concentration of sodium chloride (NaCl) on the recovery of BLIS were also assessed in this study.

## 2. Results

### 2.1. Antimicrobial of BLIS from L. bulgaricus FTDC 1211

**Figure 1:**
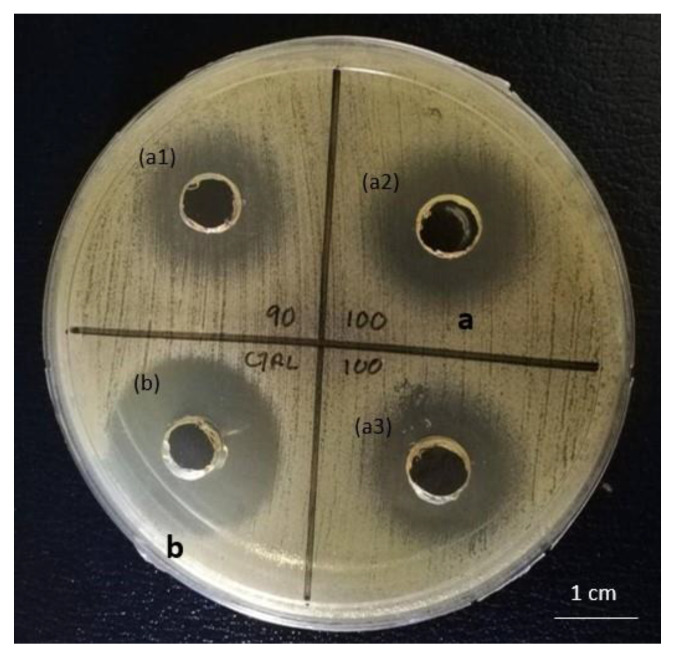
Antimicrobial activity of (**a1**) 90% (*v*/*v*) of CCFS from *L. bulgaricus* FTDC1211, (**a2**,**a3**) 100% (*v*/*v*) of CCFS from *L. bulgaricus* FTDC1211 in duplicate and (**b**) control with 15 mg/mL of streptomycin against *S. aureus*.

Figure 1 shows that the average diameter of the inhibition zone formed in the presence of CCFS from *L. bulgaricus* FTDC 1211 was 18.34 mm, contributing 2056.4 mm^2^/mL of inhibition of *S. aureus*. The BLIS activity was comparable to that of the control (15 mg/mL of streptomycin) (2102.5 mm^2^/mL). Hence, the CCFS from *L. bulgaricus* FTDC 1211 was used for subsequent experiments.

### 2.2. Purification of BLIS from L. bulgaricus FTDC 1211 Using AIRS

#### 2.2.1. Effect of Molecular Weight and Concentration of PEG

BLIS produced by *L. bulgaricus* FTDC 1211 was purified via a single-step AIRS. The effect of the molecular weight and concentration of PEG on purification of BLIS by AIRS is shown in Table 1. The results showed that 10% of PEG 4000 impregnated Amberlite XAD4 resins had a higher purification factor (1.64) and recovery yield (82.63%) than other molecular weights and concentrations of PEG. It was observed that an increased concentration of PEG decreased the purification factor. The recovery of BLIS in PEG 4000 and PEG 8000 reduced markedly when the concentration of PEG increased from 10% to 40%. Hence, 10% (*w*/*w*) of PEG 4000 with the highest purification factor was used in further experiments.

#### 2.2.2. Effect of Sodium Citrate Concentration

**Figure 2:**
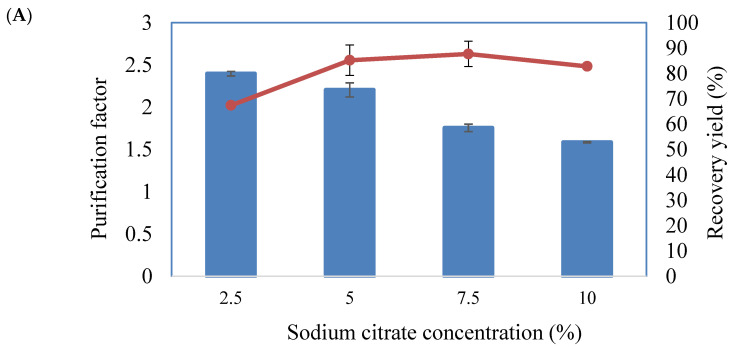

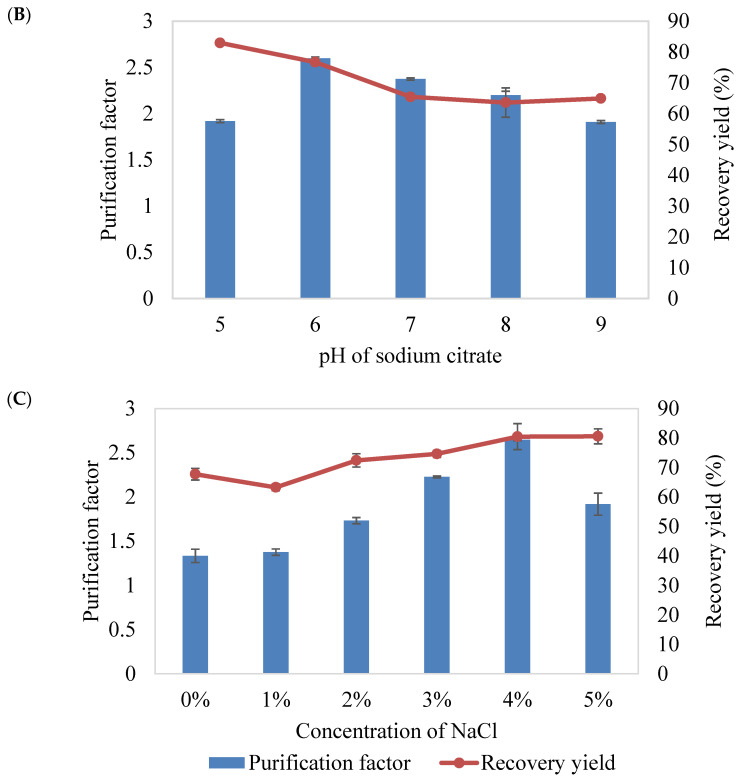
The purification factor and recovery yield achieved for different concentration of sodium citrate (**A**), pH of sodium citrate (**B**) and NaCl (**C**). The results reported were expressed as a mean of triplicate reading with an estimated error of ±5%.

Figure 2A shows the effect of the sodium citrate concentration (2.5, 5, 7.5 and 10% (*w*/*w*)) in the extraction solution on the purification factor and yield of BLIS in AIRS. The highest purification factor (2.4) was obtained with 2.5% sodium citrate. However, the recovery of BLIS was reduced to 67.4%. The purification factor showed a decreasing trend as the concentration of sodium citrate increased. Hence, 2.5% (*w*/*w*) of sodium citrate was used in subsequent experiments.

#### 2.2.3. Effect of pH of Sodium Citrate

Figure 2B shows the effect of the pH of sodium citrate on the purification factor and recovery yield by manipulating the charges of the protein in the system. Among five different pHs of sodium citrate (5, 6, 7, 8 and 9), a pH of 6 showed the highest purification factor (2.6) and recovery yield (76.7%). As the pH increased from 6 to 9, the purification factor gradually reduced.

#### 2.2.4. Effect of Different Concentration of NaCl

The effect of NaCl concentration on purification factor and recovery yield of BLIS is presented in Figure 2C. The addition of 4% (*w*/*w*) NaCl into the extraction solution showed the highest purification factor (2.65) and recovery yield (80.67%). When the NaCl concentration increased to 4% (*w*/*w*), the purification factor gradually increased. Further increasing the NaCl concentration resulted in a decrease in purification factor.

### 2.3. Optimization of Purification Parameters Using Response Surface Methodology

A five level, full factorial central composite design (CCD) with three independent variables (concentration of PEG, pH of sodium citrate and concentration of sodium citrate) was applied to maximize the recovery of BLIS in AIRS. The actual and predicted purification factor and recovery yield (%) are presented in Table 2. In this study, the maximum purification factor (2.98 ± 0.03) and recovery yield (84.28% ± 0.06) were predicted to be obtained under optimum conditions, i.e., 11.44% (*w*/*w*) PEG 4000 and 1.98% (*w*/*w*) sodium citrate at a pH of 6.06.

An analysis of variance (ANOVA) for the purification factor and recovery yield was performed based on the experimental data presented in Table 3. Fisher’s *F*-test for purification factor showed that the model was capable of representing the relationships between the purification factor (response) with the three selected factors (concentration of PEG, pH of sodium citrate and concentration of sodium citrate). The *F*-value for the purification factor of model (32.90) with a *p*-value of <0.0001 indicated that the model is significant at 95% confidence level. Meanwhile, the *p*-value for lack of fit for purification factor model (0.3904) implied that the model is not significantly relative to pure error. The *p*-value for the interaction between PEG concentration and salt (*p*-value = 0.0002), PEG concentration and salt concentration (*p*-value = 0.0028) and pH of salt and salt concentration (*p*-value = 0.0002) indicated that the interaction between the respective parameters affected the purification factor of BLIS. On the other hand, the *F*-value for the recovery yield was 2.56 with *p*-value > 0.1001, indicating that the model is not significant at a 95% confidence level. Hence, there is no fitted model for the recovery yield. This could be due to insignificant variations in the recovery yield for low or high levels of factors.

From the statistical analysis, it was found that the most suitable fitted model for the purification factor was quadratic polynomial. The predicted response for the purification factor is in Equation (1):

Y_1_ = 2.84 − 0.43A + 0.093B − 0.20C − 0.50 AB + 0.34AC + 0.52BC − 0.74A^2^ − 0.35B^2^ − 0.30C^2^ (1)

A = PEG concentration (% *w*/*w*), B = pH of sodium citrate, C = concentration of sodium citrate (% *w*/*w*).

**Figure 3:**
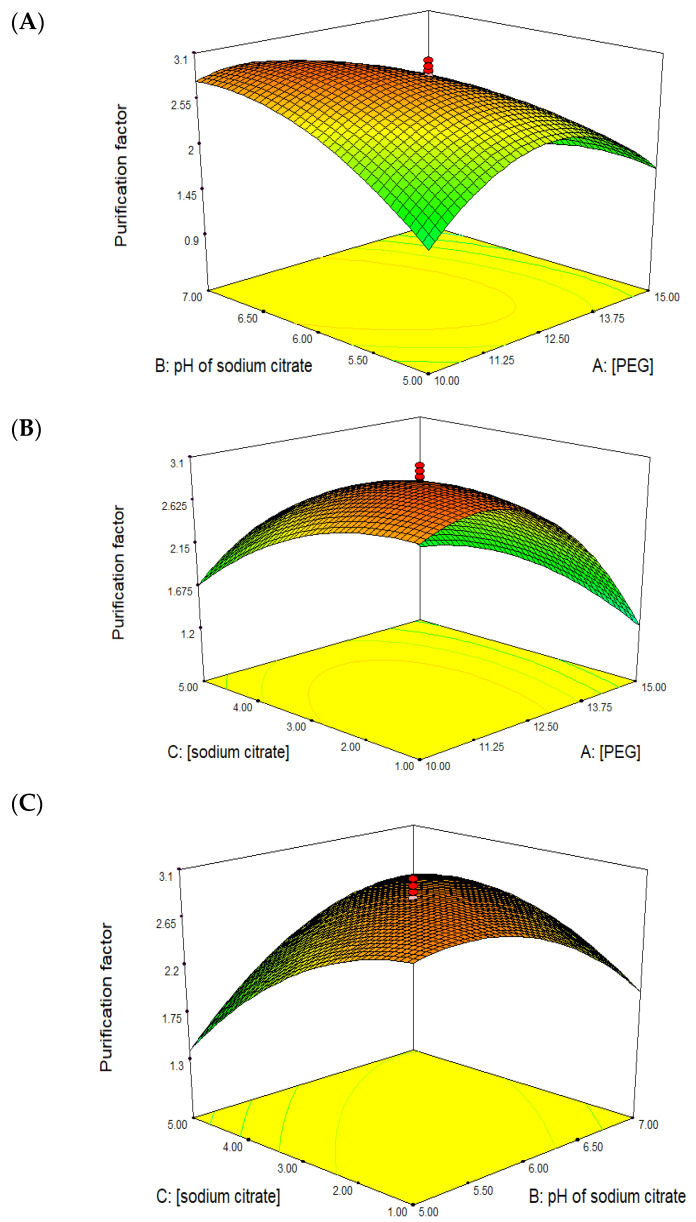
3D surface plot for purification factor obtained for the combination effect of (**A**) concentration of PEG 4000 and pH of sodium citrate, (**B**) concentration of PEG 4000 and sodium citrate concentration and (**C**) pH and concentration of sodium citrate.

Figure 3 shows the 3D surface plots for the interaction between PEG concentration and pH (A), PEG concentration and salt concentration (B) and salt concentration and pH (C). A quadratic polynomial curve with an optimum point was obtained from the interaction between the parameters. Validation of the experimental data showed that the purification factor of BLIS (3.25 ± 0.03) had small variation relative to the predicted purification factor (2.98) under optimum purification conditions, i.e., 11.4% (*w*/*w*) of PEG 4000 impregnated on Amberlite XAD-4 resins, 2% (*w*/*w*) of sodium citrate at pH 6 and 4% (*w*/*w*) of NaCl.

### 2.4. Purity of BLIS on SDS-PAGE

**Figure 4:**
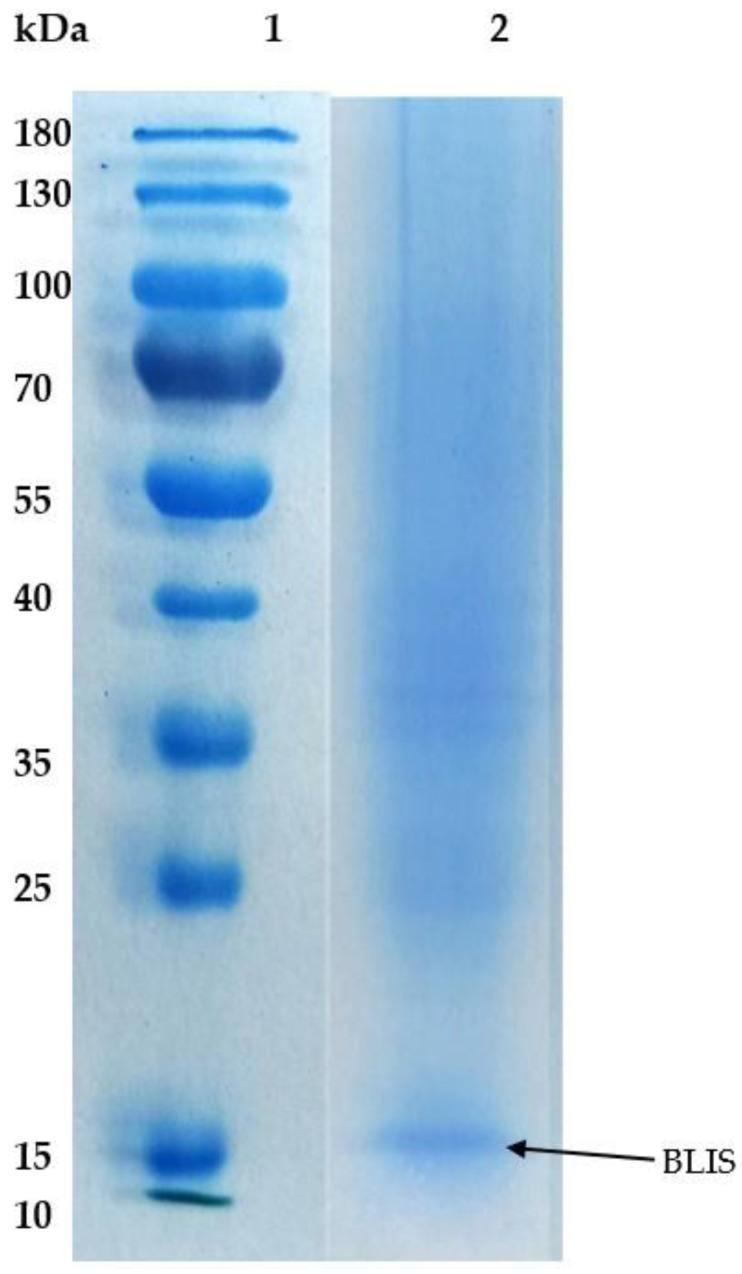
SDS-PAGE profile. Lane 1: standard marker with molecular weight of 10 to 180 kDa. Lane 2: partially purified BLIS from *L. bulgaricus* FTDC1211.

The purified BLIS was analyzed using 12% SDS-PAGE image gel to determine the purity. Figure 4 shows that the BLIS was partially purified by AIRS. There is a band with an approximate molecular size of 14 kDa in Lane 2 which was assumed to be the BLIS that had been partially purified from *L. bulgaricus* FTDC 1211 by AIRS.

## 3. Discussion

In order to confirm the antimicrobial effect of the CCFS on *S. aureus* ATCC12600, a well diffusion assay was used to determine the inhibitory activity of *L. bulgaricus* FTDC 1211. The results showed that *L. bulgaricus* FTDC 1211 can inhibit the growth of *S. aureus*. There is a consensus regarding the hypothesis that most bacteriocins interact with cell membrane anionic lipids of the target bacteria, causing their permeabilization through the formation of pores. Eventually, this interaction can cause the death of the target cell, promoting the dissipation of the proton motive force (PMF) and the inhibition of amino acids transport. PMF is involved in several processes in the cell membrane, such as the accumulation of ions and metabolites, and ATP synthesis [10].

BLIS produced by *L. bulgaricus* FTDC 1211 was purified via a single-step AIRS. The results from the effect of molecular weight and concentration of PEG on the purification of BLIS by AIRS revealed that the PEG molecular weight had an effect on the purification of BLIS in AIRS. As the PEG mass increased from 2000 to 4000, the surface hydrophobicity increased in the PEG impregnated Amberlite XAD-4 resins due to the reduction of the hydroxyl groups for the higher chain length PEG, as compared to the lower chain length [9,11]. Hence, BLIS with a hydrophobic surface area were selectively adsorbed into the PEG impregnated resins, subsequently increasing the purification factor and recovery yield. On the other hand, increase in PEG mass led to an increase of excluded volume, in which there was less space to accommodate protein in PEG impregnated Amberlite XAD-4 resins. Hence, the purification factor decreased with increasing the PEG molecular weight to 6000 and 8000 due to the excluded volume effect. Sabrina da Silva Sabo et al. [12] reported that a low concentration of PEG could increase the purification factor of BLIS in an aqueous two phase system. Hence, 10% (*w*/*w*) of PEG 4000 with the highest purification factor was used in further experiments.

The results from the effect of the sodium citrate concentration on the purification factor and yield of BLIS in AIRS demonstrated that there was a decreasing trend in the purification factor as the sodium citrate concentration increased. This could be attributed to the higher concentration of salt, which led to a salting-out effect of the protein molecule [13]. The salting-out effect caused the precipitation of the BLIS in the bulk salt phase and prevented it from binding to PEG impregnated resins. Unlike ATPS, a high concentration of salt is usually needed for the phase formation in AIRS, as it could increase the interfacial tension and density difference between the PEG and salt phases [8,14]. Hence, 2.5% (*w*/*w*) sodium citrate was selected for use in subsequent experiments.

The effect of pH of sodium citrate on the purification factor and recovery yield was due to the manipulation of the protein charges in the system. As the pH increased from 6 to 9, the purification factor gradually decreased. This could be due to the effect of pH on the charge of the biomolecules, their ion composition and the surface character of contaminating materials, which resulted in a variation in the biomolecule extraction by the resins. The pH of the salt could affect the extraction of the targeted protein, in which the adsorption of biomolecule markedly increased when the protein charge changed from positive to neutral [15]. There was no net molecular charge for proteins in a solution with the same pH of their pI. Electrostatic repulsion between protein molecules would then be at a minimum and interactions via hydrophobic groups on the surface of the protein would occur [16]. Therefore, the pH of the system may be manipulated in order to promote selective separation.

Increasing the NaCl concentration from 0 to 4% (*w*/*w*) gradually increased the purification factor. However, further increase in NaCl concentration resulted in a decrease in the purification factor. The addition of NaCl increased the difference in the hydrophobicity of PEG and sodium citrate, causing more BLIS to be extracted to the PEG impregnated resins [8]. In this study, the extraction and back-extraction of the target biomolecule was driven by the presence of a neutral salt, i.e., NaCl. NaCl is commonly used in PEG-salt ATPS to alter the partitioning behavior of proteins by distributing differential to the salt ions between the polymer and salt [11].

BLIS was partially purified by AIRS with approximate molecular size of 14 kDa. Based on previous findings by Nour I. et al. [17] and Kim et al. [18], the molecular weights of BLISs produced from *L. bulgaricus* were 14.4 kDa and 45 kDa. In a study by Balogu et al. [19], the CCFS of *L. bulgaricus* Y34, purified using ammonium sulphate precipitation, TCA precipitation, followed by ultrafiltration, had 8.8% bacteriocin recovery with a P_FT_ of 11.8 at the end of the purification process. The ammonium sulfate and TCA precipitation processes in their study yielded 17.5% bacteriocin recovery and a P_FT_ of 5.7. Higher P_FT_ could be observed after the precipitation steps. Hence, the precipitation steps could be used in our study to strengthen the purification performance

## 4. Materials and Methods

### 4.1. Supplies

Polyethylene glycols (PEGs) with an average molecular weight of 2000 g/mol, 4000 g/mol and 6000 g/mol, nutrient agar and nutrient broth were purchased from Merck (Darmstadt, Germany) while PEG 8000 g/mol was obtained from OmniPur Plus (Darmstadt, Germany). Potassium citrate was sourced from HmbG (Hamburg, Germany). De Man Ragosa and Sharpe (MRS) broth, Amberlite XAD4 with pore size distribution of ~0.98 mL/g pore volume, sodium chloride, acetone, acrylamide/bis-acrylamide (30% solution), ammonium persulphate were supplied from Sigma Aldrich (St. Louis, MO, USA). 0.5M Tris-HCl buffer (pH 6.8), 1.5 M Tris-HCl buffer (pH 8.8), sodium dodecyl sulphate, tetramethylenediamine (TEMED) and concentrated Bradford reagent were purchased from Bio-Rad (Hercules, CA, USA). PageBlue Protein Staining solution was obtained from Thermo Scientific (Waltham, MA, USA).

### 4.2. Bacteriocin-Producing LAB and BLIS Preparation

A BLIS producing strain, *L. bulgaricus* FTDC 1211 isolated from cow milk was obtained from Bioprocess Technology laboratory, School of Industrial Technology, Universiti Sains Malaysia. The primary culture was prepared by inoculating 1 mL of bacterial stock culture with 9 mL of MRS broth and incubated at 37 °C for 24 h. The cultivation of *L. bulgaricus* FTDC 1211 was carried out with inoculation of 1 mL of inoculum into 9 mL of sterile MRS broth containing 2% (*w*/*v*) of sucrose and incubated at 37 °C for 72 h under anaerobic condition. After 72 h, the culture was centrifuged (Centrifuge Model 5500, Kubota, Japan) at 10,000 rpm, for 20 min at 4 °C and the cell culture free supernatant (CCFS) was kept at −20 °C prior to use.

### 4.3. Purification of BLIS

In our preliminary study, VitraPor porous glass beads with diameter of 4.0 mm and 8.0 mm were used. The result showed that Amberlite XAD4 resins had higher adsorption performance towards BLIS in AIRS (Data not shown). Hence, XAD4 was chosen for the present study. The impregnation of the PEG into Amberlite XAD4 resins was carried out using the dry impregnation method, as described by Abdul Aziz [9]. A total of 1 g (*w*/*w*) of PEG was needed to fully impregnate 1g of Amberlite XAD4 resins. The crude BLIS was mixed with sodium citrate and 4% NaCl at a ratio of 1:1. The mixture (2.5 mL) was loaded to the impregnated Amberlite XAD4 resins and equilibrated for 30 min. Back extraction was performed using 2.5 mL of sodium citrate in the absence of NaCl. All experiments were performed in triplicate, and the back-extracted fraction was assessed for antimicrobial activity.

Furthermore, the effect of molecular weight (2000 to 8000 g/mol) and concentration (10 to 40%, *w*/*w*) of PEGs, concentration of sodium citrate solution (2.5 to 10%, *w*/*w*), pH of sodium citrate solution (5 to 9) and concentration of NaCl (1 to 5%, *w*/*w*) on the purification of BLIS were studied by using one-factor-at-a-time method. The significant parameters were further optimized using response surface methodology. A five-level, full-factorial central composite design (CCD) was designed using Design-Expert (version 7.1.6, Stat-Ease Inc., Minneapolis, MN, USA) for regression modelling and data interpretation. The observed responses from CCD design were then fitted to the polynomial Equation (2)

(2) $$Y=\beta_{0}+\overset{k}{\underset{i=1}{\sum }}\beta_{i}x_{i}+\overset{k}{\underset{i=1}{\sum }}\beta_{ii}x_{i}^{2}+\overset{k}{\underset{i=1}{\sum }}\overset{k}{\underset{j=1}{\sum }}\beta_{ij}x_{i}\beta_{ii}x_{j}+\varepsilon$$

where *Y* is the predicted response; *i* and *j* are the index numbers for the pattern; *β* is the offset term; *β_i_*, *β_ii_*, and *β_ij_* are the coefficients for the linear, quadratic, and interaction effects, respectively; *x_i_* and *x_j_* are the coded variables; and *ε* is the error.

The regression equation was optimized by an iterative method to achieve the optimum values. From this step, the optimum parameters with highest desirability in purification factor were determined.

### 4.4. Antimicrobial Activity Assay

The antimicrobial activity of BLIS from *L. bulgaricus* FTDC 1211 was evaluated using well-diffusion assay as described by Ng et al. [20]. *S. aureus* ATCC 12600 was cultured overnight in nutrient broth and agitated at 150 rpm, at 30 °C. The culture was adjusted to 1−2 × 10^6^ CFU/mL. A prepared nutrient agar was inoculated with *S. aureus* using a cotton swab, and a well was made on the agar plate. Then, 100 µL of sample was pipetted into the well. Streptomycin with dose of 15µg was used as positive control. The nutrient agar plate was incubated at 37 °C for 24 h. The diameter of the clear zone was recorded and the BLIS activity was calculated using Equation (3):

(3) $$\mathrm{BLIS}\;\mathrm{activity}\;({\mathrm{mm}}^{2}/\mathrm{mL})=\frac{\left(L2-L1\right)}{V}$$

where *L*2 and *L*1 are the diameters of clear zone and well zone, respectively, and *V* is the volume of CCFS.

### 4.5. Total Protein Content Determination

The total protein content was determined using Bradford method [21]. Briefly, 10 µL of sample was mixed with 200 µL of diluted concentrated Bradford dye reagent (1 mL of dye reagent diluted with 4 mL of distilled water) in a 96-well microplate. The samples were incubated at room temperature for 10 min before reading the absorbance at 595 nm using a microplate reader (Halo MPR-96 Visible Microplate Reader, Dynamica). Bovine Serum Albumin (BSA) at a concentration range of 0.0625 to 1.0 mg/mL was used as standard curve and the total protein content was calculated using BSA standard curve. All the experiments were performed in triplicate.

### 4.6. Sodium Dodecyl Sulfate Polyacrylamide Gel Electrophoresis

Prior to SDS-PAGE, BLIS was precipitated using trichloroacetic acid (TCA). First, 200 µL of TCA stock solution was added to the 800 µL sample and incubated in 4 °C for 15 min before centrifugation at 13,000 rpm for 10 min. The pellet was then washed with 200 µL of cold acetone. The washing steps were repeated three times and the pellet were dried in a drying cabinet at 60 °C for 30 min. SDS-PAGE of the purified BLIS was performed using 12% resolving gel and 4% stacking gel. Then, 100 µL of Laemmli sample buffer was added to the pellet, which was heated at 95 °C for 15 min. Next, 15 µL of samples and 5 µL of marker (size range from 10 to 180 kDa) were loaded into the well and the electrophoresis was performed at a constant voltage of 120 V for 60 min. The gel was stained overnight using PageBlue protein staining solution and destained using a solution containing 10% of methanol and 10% of acetic acid overnight. The image of the band was subsequently captured using gel imaging system (UV Tech Gel Imaging System, Vivantis, Malaysia).

### 4.7. Determination of Purification Factor and Recovery Yield

The specific activity (SA), purification factor (P_FT_) and recovery yield of BLIS were calculated using the following equations.

The SA defined as the ratio between the depleted BLIS antimicrobial activity (mm^2^/mL) and the concentration of the total protein content were calculated using Equation (4):

(4) $$\mathrm{SA}\;({\mathrm{mm}}^{2}/\mathrm{mg})\;=\;\frac{\mathrm{BLIS}\;\mathrm{antimicrobial}\;\mathrm{activity}\;\left(\frac{{\mathrm{mm}}^{2}}{\mathrm{mL}}\right)}{\left[\mathrm{total}\;\mathrm{protein}\right]\left(\frac{\mathrm{mg}}{\mathrm{mL}}\right)}$$

P_FT_ is defined as the ratio of the SA in the depleted BLIS to SA in crude BLIS was calculated based on Equations (5) and (6), respectively.

(5) $$\mathrm{Purification}\;\mathrm{factor},\;P_{\mathrm{FT}}\;=\;\frac{\mathrm{SA}\;\mathrm{in}\;\mathrm{back}\;\mathrm{extraction}\;\mathrm{solution}\;\left(\frac{{\mathrm{mm}}^{2}}{\mathrm{mg}}\right)}{\mathrm{SA}\;\mathrm{in}\;\mathrm{crude}\;\mathrm{BLIS}\;\left(\frac{{\mathrm{mm}}^{2}}{\mathrm{mg}}\right)}$$

The recovery yield of the BLIS was estimated using Equation (5):

(6) $$\mathrm{Recovery}\;\mathrm{yield}\;(\%)=\frac{\begin{array}{c} \mathrm{BLIS}\;\mathrm{antimicrobial}\;\mathrm{activity}\;\mathrm{in}\;\\ \mathrm{back}\;\mathrm{extraction}\;\mathrm{solution} \end{array}}{\begin{array}{c} \mathrm{BLIS}\;\mathrm{antimicrobial}\;\\ \mathrm{activity}\;\mathrm{in}\;\mathrm{crude}\;\mathrm{BILS} \end{array}}\times 100$$

## 5. Conclusions

BLIS from *L. bulgaricus* FTDC 1211 was partially purified in this study. The partially purified BLIS with an estimated size of 14 kDa and total recovery of 82.96% with a purification factor of 3.26 was achieved under the optimum condition of 11.4% (*w*/*w*) PEG 4000 impregnated on Amberlite XAD4 and 2% (*w*/*w*) sodium citrate at pH 6.06 with 4% (*w*/*w*) NaCl. These results demonstrate that AIRS could be used as an alternate purification system in the primary recovery step. The BLIS purification method from fermentation broth described in this study need to be further improved. Regarding the use of the AIRS, additional purification methods such as ultrafiltration, anion exchange chromatography, gel-filtration for reduction of the contaminants, and HPLC or FPLC chromatography could be utilized. The purified BLIS can be sent for N-terminal sequencing. Degenerative primers based on the amino acid sequences can then be synthesized. The gene cluster could be amplified by PCR and the ORFs in the gene cluster could be analyzed and compared with the known bacteriocin. Even though AIRS are capable of purifying BLIS, there are however during the impregnation of polymer into Amberlite XAD4, leaching of the polymer occurs. Study on the mechanism of leached PEG will deepen our understanding of the system. Furthermore, study of the adsorption kinetics during the extraction of BLIS is required to understand the system. In addition, the repetition of the flow-through and recyclability of the system could be further investigated to improve the applicability of the system.

## Figures and Tables

**Table 1:** The purification factor and recovery yield of different molecular weights and concentrations of PEG.

PEG Molecular Weight g/mol	Concentration % (*w*/*w*)	Purification Factor	Recovery Yield %
2000	10	0	0
	20	1.15	81.79
	30	1.15	84.89
	40	1.20	84.25
4000	10	1.64	82.63
	20	1.40	86.68
	30	1.10	65.50
	40	0.77	47.83
6000	10	1.16	55.54
	20	1.10	78.16
	30	1.06	84.46
	40	1.00	86.13
8000	10	1.30	80.41
	20	0.82	69.10
	30	0.71	72.38
	40	0.62	62.38

**Table 2:** The actual and predicted purification factor and recovery yield of BLIS in AIRS with different PEG concentration, pH and concentration of sodium citrate.

PEG Concentration	pH of Sodium Citrate	Concentration of Sodium Citrate	Purification Factor		Recovery Yield (%)	
			Actual	Predicted	Actual	Predicted
10.00	5.00	1.00	2.46	2.33	86.32	91.87
15.00	5.00	1.00	1.79	1.80	88.02	82.62
10.00	7.00	1.00	2.44	2.48	86.58	95.34
10.00	5.00	5.00	0.31	0.23	88.44	35.94
15.00	5.00	5.00	1.17	1.05	78.54	94.72
10.00	7.00	5.00	2.53	2.44	83.36	49.01
15.00	7.00	5.00	1.22	1.27	83.87	42.70
8.30	6.00	3.00	1.33	1.46	91.24	49.16
12.50	4.32	3.00	1.52	1.69	92.34	95.26
12.50	7.68	3.00	2.03	2.00	83.87	73.35
12.50	6.00	-0.36	2.29	2.31	84.38	91.07
12.50	6.00	6.36	1.54	1.65	80.57	89.08
12.50	6.00	3.00	3.00	2.84	82.35	82.21
12.50	6.00	3.00	2.87	2.84	82.60	82.21
12.50	6.00	3.00	2.83	2.84	85.65	82.21
12.50	6.00	3.00	2.50	2.84	83.87	82.21
12.50	6.00	3.00	2.88	2.84	83.7	82.21
12.50	6.00	3.00	2.94	2.84	81.58	82.21
Predicted optimum conditions						
11.44	6.06	1.98	3.25 ± 0.03	2.98	82.69 ± 0.06	84.28

**Table 3:** Analysis of variance (ANOVA) for response surface quadratic model of purification factor (a) and recovery yield (b) of BLIS.

Source	Sum of Squares	Degree of Freedom	Mean Square	*F* Value	*p*-Value Prob > *F*
**(a) Purification Factor**					
Model	9.82	9	1.09	32.90	<0.0001 ^a^
A	0.97	1	0.97	29.18	0.0006
B	0.089	1	0.089	2.69	0.1393
C	0.40	1	0.40	11.97	0.0086
AB	1.30	1	1.30	39.31	0.0002
AC	0.60	1	0.60	18.12	0.0028
BC	1.39	1	1.39	41.85	0.0002
A^2^	3.49	1	3.49	105.21	<0.0001
B^2^	1.70	1	1.70	51.15	<0.0001
C^2^	1.25	1	1.25	37.78	0.0003
Residual	0.27	8	0.033		
Lack of fit	0.11	3	0.038	1.23	0.3904 ^b^
Pure error	0.15	5	0.031		
Cor Total	10.08	17			
**(b) Recovery Yield**					
Model	157.69	9	17.52	2.56	0.1001 ^b^
A	5.07	1	5.07	0.74	0.4145
B	9.44	1	9.44	1.38	0.2740
C	26.68	1	26.68	3.90	0.0838
AB	9.65	1	9.65	1.41	0.2691
AC	14.34	1	14.34	2.09	0.1859
BC	0.044	1	0.044	6.485 × 10^−3^	0.9378
A^2^	19.07	1	19.07	2.79	0.1336
B^2^	28.60	1	28.60	4.18	0.0752
C^2^	4.04	1	4.04	0.59	0.4645
Residual	54.75	8	6.84		
Lack of fit	44.40	3	14.80	7.14	0.0295 ^a^
Pure error	10.36	5	2.07		
Cor Total	212.45	17			

A = PEG concentration (% *w*/*w*), B = pH of sodium citrate, C = concentration of sodium citrate (% *w*/*w*), ^a^ Significant and ^b^ Not significant.
